# Determinants and Temporal Dynamics of Cerebral Small Vessel Disease: 14-Year Follow-Up

**DOI:** 10.1161/STROKEAHA.121.038099

**Published:** 2022-05-04

**Authors:** Mengfei Cai, Mina A. Jacob, Mark R. van Loenen, Mayra Bergkamp, José Marques, David G. Norris, Marco Duering, Anil M. Tuladhar, Frank-Erik de Leeuw

**Affiliations:** Department of Neurology, Radboud University Medical Center (M.C., M.A.J., M.B., A.M.T., F.-E.d.L.), Donders Institute for Brain, Cognition and Behaviour; Nijmegen, the Netherlands.; Center for Cognitive Neuroimaging (M.R.v.L., J.M., D.G.N.), Donders Institute for Brain, Cognition and Behaviour; Nijmegen, the Netherlands.; Medical Image Analysis Center (MIAC AG) and qbig, Department of Biomedical Engineering, University of Basel, Switzerland (M.D.).

**Keywords:** cerebral small vessel disease, magnetic resonance imaging, neuroimaging, risk factor, white matter hyperintensities

## Abstract

The aim of this study is to investigate the temporal dynamics of small vessel disease (SVD) and the effect of vascular risk factors and baseline SVD burden on progression of SVD with 4 neuroimaging assessments over 14 years in patients with SVD.

**Methods:**

Five hundred three patients with sporadic SVD (50–85 years) from the ongoing prospective cohort study (RUN DMC [Radboud University Nijmegen Diffusion Tensor and Magnetic Resonance Cohort]) underwent baseline assessment in 2006 and follow-up in 2011, 2015, and 2020. Vascular risk factors and magnetic resonance imaging markers of SVD were evaluated. Linear mixed-effects model and negative binomial regression model were used to examine the determinants of temporal dynamics of SVD markers.

**Results:**

A total of 382 SVD patients (mean [SD] 64.1 [8.4]; 219 men and 163 women) who underwent at least 2 serial brain magnetic resonance imaging scans were included, with mean (SD) follow-up of 11.15 (3.32) years. We found a highly variable temporal course of SVD. Mean (SD) WMH progression rate was 0.6 (0.74) mL/y (range, 0.02–4.73 mL/y) and 13.6% of patients had incident lacunes (1.03%/y) over the 14-year follow-up. About 4% showed net WMH regression over 14 years, whereas 38 out of 361 (10.5%), 5 out of 296 (2%), and 61 out of 231 (26%) patients showed WMH regression for the intervals 2006 to 2011, 2011 to 2015, and 2015 to 2020, respectively. Of these, 29 (76%), 5 (100%), and 57 (93%) showed overall progression across the 14-year follow-up, and the net overall WMH change between first and last scan considering all participants was a net average WMH progression over the 14-year period. Older age was a strong predictor for faster WMH progression and incident lacunes. Patients with mild baseline WMH rarely progressed to severe WMH. In addition, both baseline burden of SVD lesions and vascular risk factors independently and synergistically predicted WMH progression, whereas only baseline SVD burden predicted incident lacunes over the 14-year follow-up.

**Conclusions:**

SVD shows pronounced progression over time, but mild WMH rarely progresses to clinically severe WMH. WMH regression is noteworthy during some magnetic resonance imaging intervals, although it could be overall compensated by progression over the long follow-up.

Cerebral small vessel disease (SVD) is the most important vascular contributor to dementia and accounts for up to a fifth of all stroke worldwide.^1^ White matter hyperintensities (WMH) and lacunes of presumed vascular origin are hallmark lesions of SVD on magnetic resonance imaging (MRI).^2,3^ Progression of these lesions is associated with poor functional outcomes, including cognitive decline,^4^ gait dysfunction,^5–7^ and depression.^8^ Vascular risk factors and baseline severity of SVD have been identified as determinants of SVD progression.^9–11^ However, this may be different for rapid and slow progressors and for early- or late-stage disease.^12,13^ Besides, it is still unknown whether vascular risk factors and SVD lesions at baseline affect the long-term SVD changes independently or in interaction.

Emerging evidence has shown regression in some SVD patients.^10,13–15^ However, our understanding of individual temporal course of SVD MRI markers and the identification of the individuals at risk of rapid progression and regression is limited. Because most studies had a relatively short duration of follow-up (no more than 6 years) with usually only a baseline and one follow-up scan, which does not allow for the identification of regression alternated by progression.^10^

Therefore, studies with a longer follow-up and serial follow-up MRI scans are warranted to comprehensively investigate the temporal dynamics of SVD and its determinants. We investigated the temporal course of WMH and lacunes, as markers of SVD, by 4 consecutive neuroimaging assessments over 14 years in older patients with sporadic SVD. In addition, we examined the effect of vascular risk factors, SVD burden at baseline, and their interaction with the long-term temporal dynamics.

## Methods

The data that support the findings of this study are available from the corresponding author upon reasonable request. Results were reported in adherence to the STROBE statement guidelines.

### Study Population

This study is part of the RUN DMC study (Radboud University Nijmegen Diffusion Tensor and Magnetic Resonance Cohort), an ongoing longitudinal prospective single-center study that aims to investigate the risk factors and clinical consequences of sporadic SVD in people aged between 50 and 85 years, as described in the study protocol.^16^ Because the onset of cerebral SVD is often insidious, clinically heterogeneous, and typically with mild symptoms, it has been suggested that the selection of subjects with SVD in clinical studies should be based on these more consistent brain imaging features.^17^ Accordingly, in 2006, consecutive patients referred to the Department of Neurology between October 2002 and November 2006, were selected for participation. Inclusion criteria were (1) age between 50 and 85 years and (2) cerebral SVD on neuroimaging (WMHs and/or lacunes) with the accompanying acute or subacute clinical symptoms (eg, transient ischemic attack, lacunar syndromes, cognitive and motor disturbances) of SVD. Patients who were eligible because of a lacunar syndrome were included only >6 months after the event to avoid acute effects on the outcomes.

**Figure 1.**
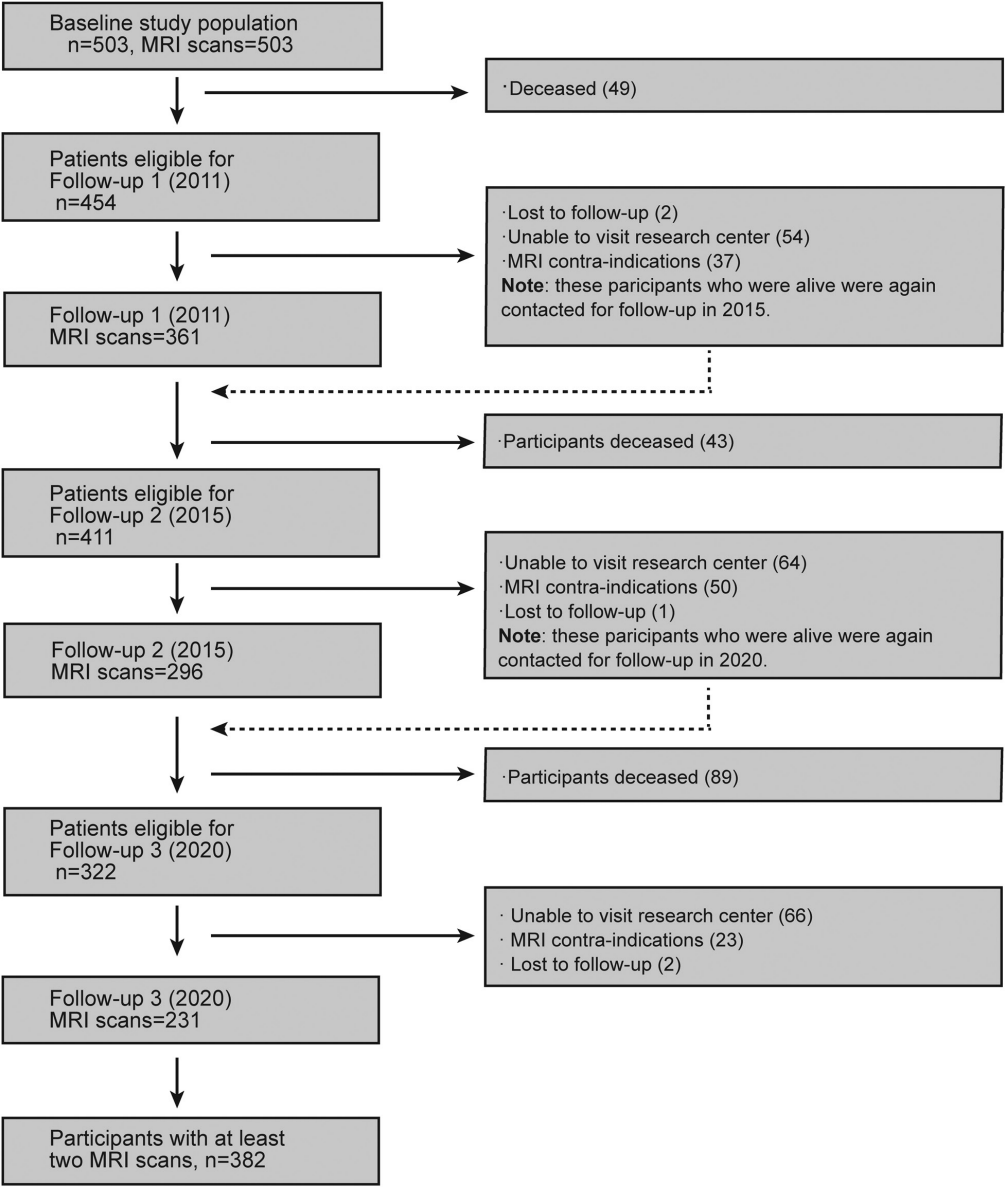
**Flow chart of RUN DMC study (Radboud University Nijmegen Diffusion Tensor and Magnetic Resonance Cohort) population over time.** MRI indicates magnetic resonance imaging.

Baseline data collection was performed in 2006 (wave 1), with 3 follow-ups (wave 2 in 2011, wave 3 in 2015, wave 4 in 2020). Because not all participants completed follow-up MRI scans, we included participants with at least one follow-up scan (n=382) to examine the temporal dynamics of SVD markers. Flowchart of RUN DMC study population over time was provided in Figure 1. Final data collection was completed on December 9, 2020. The Medical Review Ethics Committee region Arnhem-Nijmegen approved the study and all participants gave written informed consent.

### MRI Protocol

Images were acquired at wave 1, 2, 3 on 1.5-Tesla MRI (2006: Siemens, Magnetom Sonata; 2011 and 2015: Siemens, Magnetom Avanto). The same 8-channel head coil was used at all 3 time points. MRI scans at wave 4 (2020) were performed on a 3-Tesla MRI scanner (Siemens, Magnetom Prisma) with a 32-channel head coil. Detailed MRI acquisition parameters for each wave were shown in Table S1.

### MRI Processing and Brain Volumetry

At wave 1, 2, and 3, WMH was segmented semi-automatically using fluid-attenuated inversion recovery (FLAIR) and T1 sequences, as described previously.^18^ At wave 4, MP2RAGE data were processed to obtain robust T1-weighted images, reaching the best compromise between a significant decrease in noise levels in regions of low or no signal (air or skull) and a small increase in image intensity bias.^19^ WMH was segmented from registered and bias-corrected T1 and FLAIR images by using a variant of the 3-dimensional U-net deep learning algorithm.^20^ All WMH segmentations were then manually edited and cleaned from misclassified artifacts by a custom 3-dimensional editing tool written in Matlab. Further details are provided in the Supplemental Methods.

### Assessment of Baseline SVD Burden

The rating of SVD markers, that is, WMH volume, lacune count, and microbleed count at baseline, was based on STRIVE criteria (Standards for Reporting Vascular Changes on Neuroimaging).^1^ Prevalent lacunes at baseline on T1 and FLAIR scans and microbleeds on T2*-weighted MRI images at baseline were rated manually by 2 trained and experienced raters, followed by a consensus meeting blinded for clinical data. Of note, in the present study, microbleeds information at baseline was used to evaluate baseline SVD burden. However, we did not investigate the progression of microbleeds over time because microbleeds (relative to WMH and lacunes) are more sensitive to the change in field strength and acquisition protocol we encountered during our follow-ups.^21^

To increase clinical generalizability, WMH volumes were also rated semiquantitatively according to the modified Fazekas score (mild: Fazekas 0–1; moderate: Fazekas 2; severe: Fazekas 3).^22^ The severity of SVD was assessed according to the previously reported amended SVD score (0–7) based on the information on lacunes, microbleeds, and Fazekas score.^23,24^

### Progression of SVD

To facilitate the systematic and consistent identification of incident lacunes, difference images were constructed for T1 and FLAIR image modalities. To this end, we first skull stripped the images using the Brain Extraction Tool in FMRIB Software Library. All follow-up images were then registered to the baseline scans. Difference images were generated by subtracting the registered and intensity-normalized baseline T1 and FLAIR images from the corresponding T1 and FLAIR images at the follow-ups (Figure S1). Incident lacunes were defined as a hypointense voxel cluster on a uniform background.^25,26^ Total lacune count during wave 2, 3, and 4 was the sum of baseline lacune count (in 2006) and the number of incident lacunes identified during that particular follow-up.

Follow-up WMH volumes were corrected to baseline intracranial volume, so that WMH volumetric changes over time can be quantified.

### Vascular Risk Factor Score and Health Status at Baseline

We assessed the presence of hypertension, smoking, diabetes, and hypercholesterolemia by standardized assessment and questionnaires, as described previously.^16^ A concurrent risk factor score (0–4), that is, the number of risk factors, was constructed based on the presence of the aforementioned risk factors to reflect the burden of vascular risk factors at baseline.^27^

Health status was evaluated for all participants at baseline by using 36-item short-form health survey, which is a well-established self-reported questionnaire on health status.^28^

### Statistical Analysis

The baseline characteristics were presented as mean±SD for normally distributed data and median and interquartile ranges for the skewed distributed parameters. We calculated differences in baseline characteristics between those included and excluded from the analyses using *t* test, χ^2^, or Mann-Whitney *U* test where appropriate.

For subjects with >1 MRI scans, we used all MRI scans available for each individual and linear mixed-effects regression via R package lme4 to examine WMH progression over time, with random effects of intercept and slope (with respect to follow-up time in year). The mixed model is statistically intended to account for the hierarchical nature of the data imposed by repeated measurements per subject, allowing imbalance (ie, missing data) and variability in the timing of assessments. The fixed effect of time represents the average annualized change of WMH across the whole cohort, whereas random effects of intercept and slope per participant can allow for interindividual variability. To evaluate a potential nonlinear progression of WMH, we compared the model fit after additionally including quadratic polynomial terms of follow-up time using likelihood ratio test and evaluated changes in Akaike information criteria. Because quadratic polynomial terms did not improve the model fit, we did not include the quadratic term in the models. WMH regression was defined as more than 0.25 mL volume decline, as this was shown to be the smallest change that could be confirmed visually.^15^

Because our results indicated that there was no sex difference in WMH progression, we utilized the following base model with follow-up time (time=0 as the first scan), baseline age as fixed effects, and follow-up time as the random effects for each participant. We then extracted random slopes of follow-up time for each participant to calculate each individual’s WMH progression rate. To examine the effect of baseline age, vascular risk factors, and SVD burden on WMH progression over time, we additionally added specific interaction terms between time and baseline age group, vascular risk factor score, SVD score into the base model. Finally, an interaction term between risk factor score and SVD score was added to the base model.

Differences in WMH progression rate among baseline Fazekas subgroups and age subgroups were analyzed, with Kruskal-Wallis tests followed by post hoc Dunn test because WMH progression rates were not normally distributed.

To model observed lacune counts over time, we used negative binomial mixed model with glmer.nb from R lme4 package. This model was able to account for over-dispersion (the variance larger than the mean) of count data by including a dispersion parameter that relaxes the presumption of equal mean and variance to handle the distribution of count outcome with excess zeroes,^29^ for example, lacune count.

To examine the effect of baseline age, vascular risk factors, and SVD burden on incident lacunes, we used negative binomial regression model. Because our results indicated that there was no sex difference in lacune incidence, we did not incorporate sex into the models. An interaction term between risk factor score and SVD score was additionally added to investigate whether they have a synergistic effect on incident lacunes.

The proportion of missing data in the present study was zero for all independent and outcome variables, except for WMH volumes (17%) which might introduce the bias. However, subjects with higher WMH volumes are more likely not to participant in the follow-up scans, indicating WMH volumes did not miss at random. Therefore, we have addressed this bias with linear mixed-effects model, although some residual bias may still remain.

## Results

We included 382 SVD patients with at least 2 MRI scans. The mean (SD) follow-up time was 11.15 (3.32) years. Specifically, 77/106/199 participants had 2/3/4 MRI scans, with median (mean; range) follow-up time of 5.35 (6.35; 4.64–13.68)/8.8 (9.8; 8.18–14.22)/13.73 (13.73; 12.73–14.63) years between the first and last MRI scan, respectively. Included participants were younger at baseline than nonparticipants (64.1 [SD, 8.4] versus 70.6 [SD, 8.2] years; *P*<0.001), although there was no sex difference (Table 1).

**Table 1.**
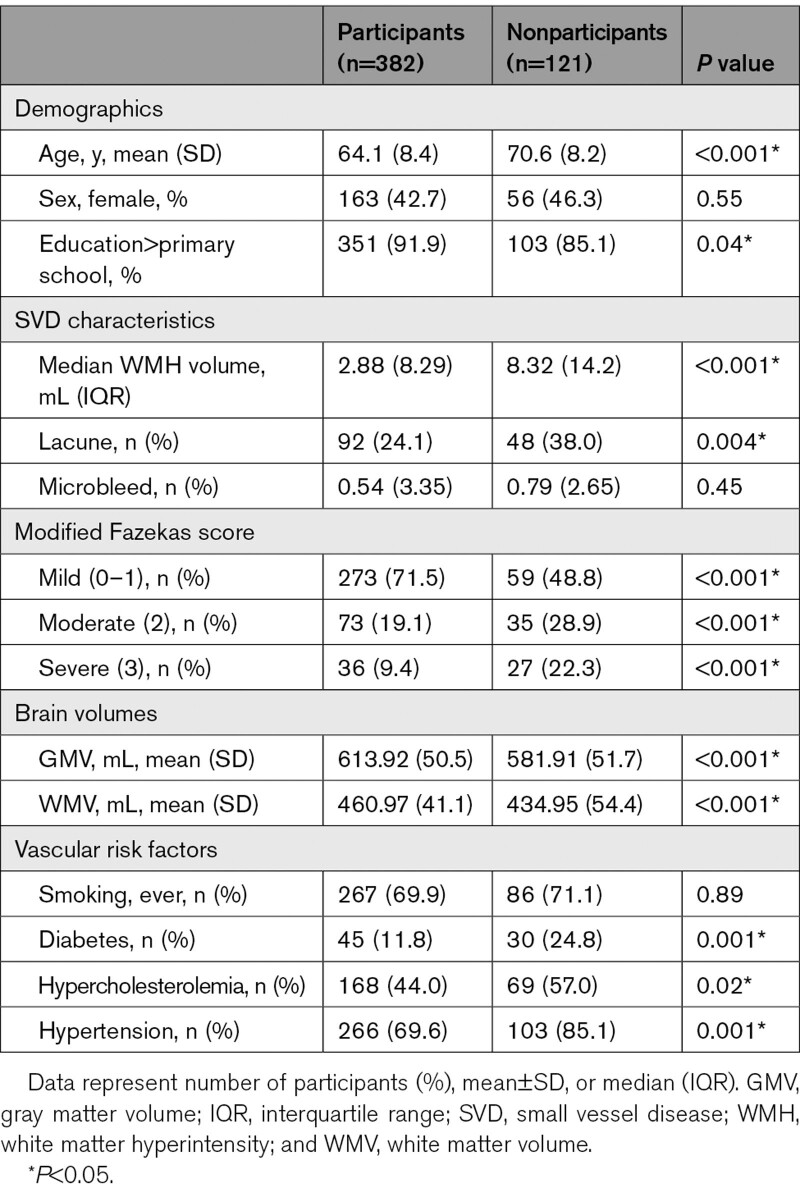
Baseline Demographics and Clinical Characteristics

### Temporal Dynamics of WMH Over Time

**Figure 2.**
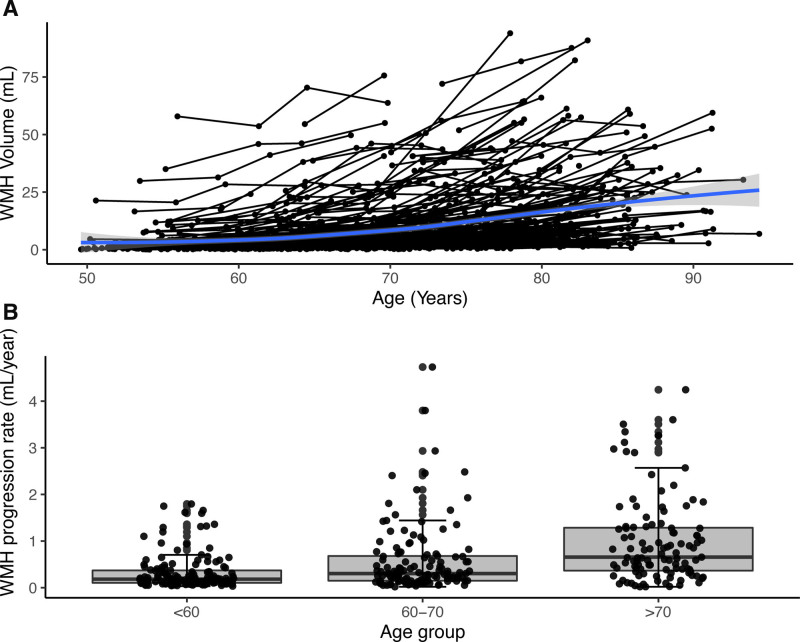
**White matter hyperintensity (WMH) trajectory and progression by age. A**, WMH trajectory over time across 4 time points at individual level. The curves were smoothed by using locally weighted smoothing (loess) to explore average WMH change with increasing age. **B**, WMH progression rate was significantly different between any 2 age subgroups at baseline, all *P*<0.001. The boxes map to the median, 25th and 75th quartiles, and whiskers extend to 1.5 × interquartile range (IQR).

Baseline age predicted WMH progression (estimate [95% CI], 0.25 [0.15–0.35]; *P*<0.001; Model 1 in Table 2, Figure 2A). The average annualized progression rate was 0.33, 0.58, 0.99 mL per year for those <60, 60 to 70, >70 years old at baseline, respectively (Figure 2B). There was a significant interaction between follow-up time and age groups (model 3 in Table 2). There was no sex difference for WMH progression (model 2 in Table 2).

**Table 2.**
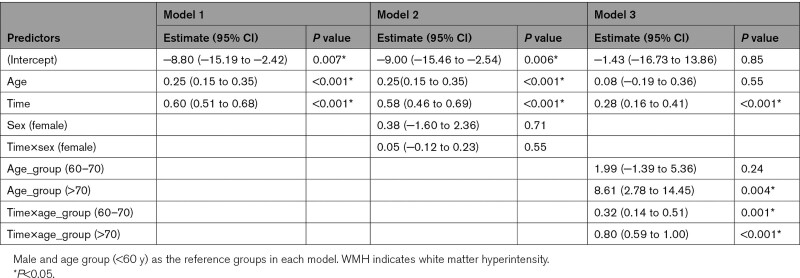
Fixed Effects Results for WMH Progression

**Figure 3.**
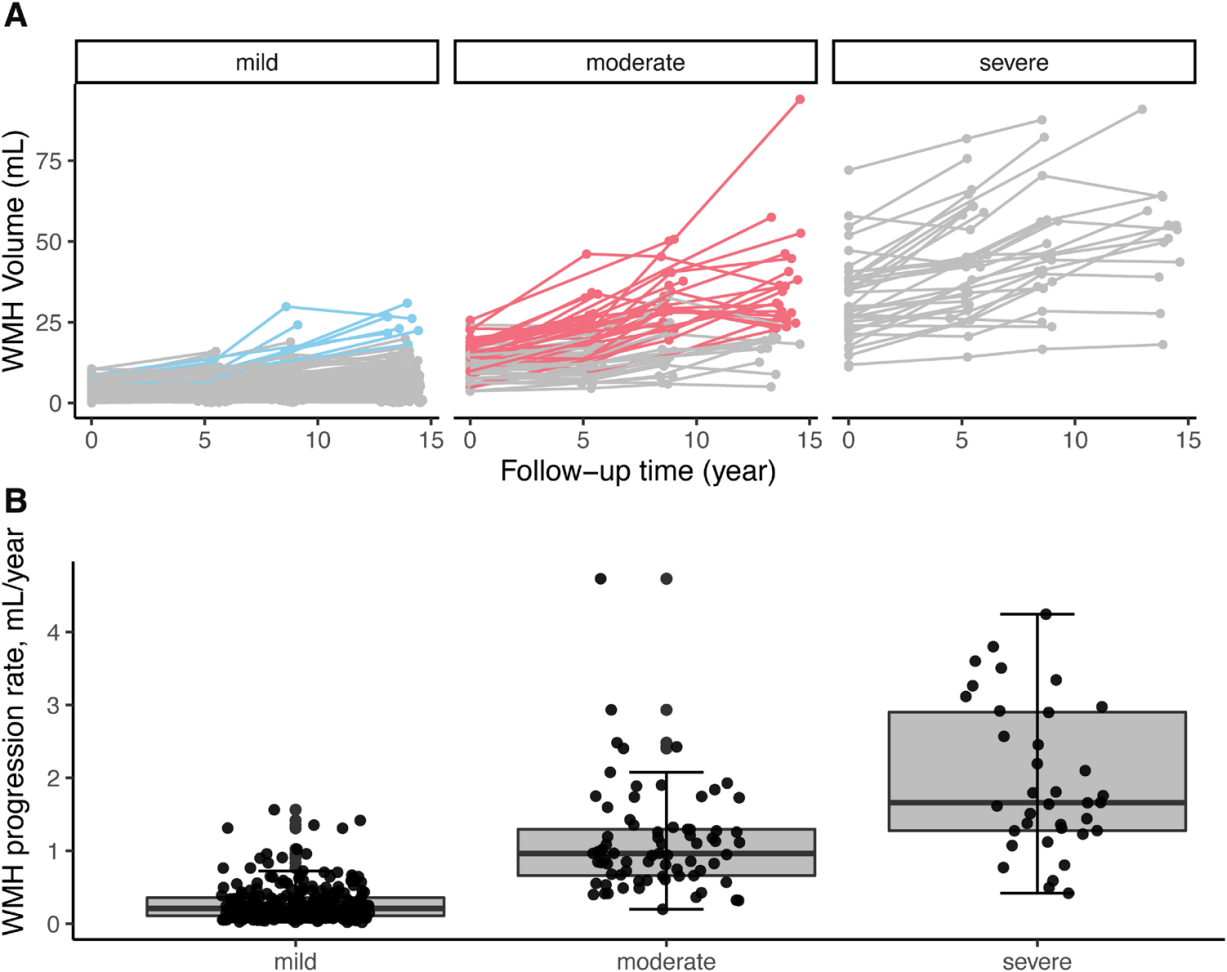
**White matter hyperintensity (WMH) progression is stratified by Fazekas groups. A**, WMH progression stratified by baseline Fazekas score. Participants who progressed into severe WMH burden with baseline mild and moderate WMH group were labeled in blue and red, respectively. **B**, WMH progression rate was significantly different between any 2 Fazekas subgroup, all *P*<0.05.

There was a significant increase in WMH volume during the 14-year follow-up. The mean (SD) yearly progression rate in the entire group was 0.6 (0.74) mL/y (range, 0.02–4.73 mL/y). Among 273 participants with mild WMH (Fazekas 0–1) at baseline, 74 (27.1%) progressed into moderate (Fazekas 2) and 8 (2.9%) into severe WMH (Fazekas 3). Among 73 participants with moderate WMH at baseline, 35 (47.9%) developed into severe WMH (Figure 3A).

### Effect of Baseline Vascular Risk Factors and SVD Burden on WMH Progression

There was a faster WMH progression in the group with ≥2 concurrent vascular risk factors compared with those without any risk factor (estimate [95% CI], 0.39 [0.08–0.70]; *P*=0.01; model 1 in Table 3), mainly driven by hypertension (Table S2). Baseline SVD burden, either measured by SVD score or Fazekas score, predicted WMH progression over time (estimate [95% CI], 0.31 [0.25–0.37]; *P*<0.001; model 2 in Table 3, Table S3). The mean WMH progression rate was 0.28, 1.12, 1.97 mL/y in the mild, moderate, severe group, respectively (Figure 3B). A significant interaction between baseline SVD score and vascular risk factor score was found (model 3 in Table 3).

**Table 3.**
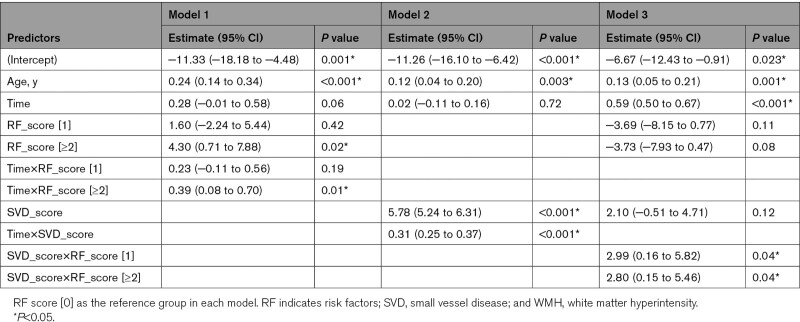
Fixed Effects Results for WMH Progression as to Baseline Vascular Risk Factors and SVD Burden Over Time

### Baseline Vascular Risk Factors and SVD Burden on Incident Lacunes

We identified a total of 92 incident lacunes in 52 patients (13.6%) between 2006 and 2020. Forty-two subjects had a single incident lacune, 4 had 2, and 6 had ≥3 incident lacunes over 14 years (average incidence rate 1.03%/y). Lacune count increased over time, independent of baseline age (estimate [95% CI], 0.08 [0.03–0.14]; *P*=0.002; Table S4).

Higher baseline SVD burden predicted incident lacunes (estimate [95% CI], 1.52 [1.27–1.85]; *P*<0.001; model 1 in Table S5). More concurrent risk factors (≥2) and their interaction with SVD burden did not predict incident lacunes. We further found that none of the vascular risk factors predicted incident lacunes (data not shown).

### WMH Regression Over Time

Regression in WMH volume was found in 15 participants (3.9%; median decline, −0.57 mL; interquartile range, 1.13 mL) during the 14-year follow-up (Figure S2, Figure S3). There were no differences in health status, any of the vascular risk factors, SVD markers, age at baseline, and rate of brain atrophy between participants with and without WMH regression (data not shown).

Regression in one interval could be compensated by progression in other time intervals as we found 38, 5, 61 patients with WMH regression for the interval 2006 to 2011, 2011 to 2015, 2015 to 2020, respectively, which was compensated by progression among 29 (76%), 5 (100%), 57 (93%) participants during the follow-ups (Figure S4).

## Discussion

In this prospective study with 14-year follow-up, we found a highly variable temporal course of SVD. Older age was a strong predictor for SVD progression, whereas sex was not. Participants with mild baseline WMH rarely progressed to severe WMH, even over 14 years. WMH regression is noteworthy during some MRI intervals, although it could be overall compensated by progression over the long follow-up. Baseline burden of SVD lesions and vascular risk factors predicted WMH progression independently and synergistically, whereas only baseline SVD burden predicted incident lacunes over the 14-year follow-up.

Several studies reported that baseline WMH severity was a strong predictor for WMH progression^12,30,31^ and incident lacunes over time.^11,32^ Our findings with much longer follow-up time not only corroborated this finding but also extended it by showing that a simple SVD score in addition to WMH grade and volume was associated with progression of conventional SVD markers. Of note, this SVD score can be assessed rapidly by visual inspection of clinical MRI scans in routine clinical practice and it has the potential to serve as a risk stratification or early efficacy assessment in clinical trials of interventions to prevent SVD progression. Besides, we also provided strong evidence that baseline mild WMH will rarely progress to severe WMH, even after 14 years. This is clinically relevant and could help SVD patients and their caregivers to put into perspective the likelihood of progression of SVD in the long term.

We demonstrated that the burden of vascular risk factors predicted WMH progression, but not incident lacunes. In addition, we found a pronounced synergistic effect between SVD burden and vascular risk factor burden on SVD progression (ie, WMH), indicative of the potential biological interplay between these factors. For example, older patients with higher WMH burden often have lower microstructural integrity in normal-appearing white matter than those with a low WMH burden.^33^ They also more often have vascular risk factors, that is, hypertension, smoking, diabetes, and hypercholesterolemia, which were found to predispose normal-appearing white matter to loss of microstructural integrity.^34–39^ Given that the development of WMH most likely is a continuous process with the impairment of WM integrity preceding MRI-visible WMH,^40^ these risk factors could may, therefore, reinforce WMH progression in interaction with baseline WMH burden. In contrast, we found that baseline risk factors were not related to incident lacunes. However, it should be noted that the association between traditional vascular risk factors and incident lacunes is largely inconclusive across different studies,^41^ which probably can be explained by different inclusion criteria and different approaches to rating incident lacunes.

Our findings have important clinical implications. First, there may be a therapeutic window earlier in life given our observation of the relation between (midlife) modifiable vascular risk factors and WMH progression in later life. Further support comes from a clinical trial showing slowing of progression of WMH in patients with intensive blood pressure management.^42^ Second, patients with mild WMH can be informed that their risk toward progression into severe WMH and the possible attendant cognitive decline is very low, even during 14 years.

Although we found evidence of WMH regression during all time intervals of the follow-up, more often regression during one interval was compensated by more progression during the other intervals. There are potentially several sources of errors for WMH measurement, for example, scanner change and differences in the acquisition protocol, image quality, and rating methods, therefore, they may influence our findings on regression. However, WMH regression may be a true biological phenomenon, given other longitudinal studies also reported WMH shrinkage in population with (minor) stroke and dementia.^14,43,44^ Biological explanations for the possible regression include that newly developed WMH may contain area of tissue edema and its subsequent resolution is likely to contribute to WMH volumetric decrease.^14^ Second, WMH on FLAIR do not only represent permanent myelin loss or axonal damage but may also represent reversible water shifts in interstitial fluid.^10^ Therefore, WMH can reduce or disappear on follow-up scans. Third, given the breakdown of blood-brain barrier was found to facilitate WMH formation, enhanced control of vascular risk factors influencing blood-brain barrier may reduce WMH volume.^45^ Of note, the WMH regression in the present study was evaluated by WMH volumes; therefore, it may not capture regression and progression in different brain areas in the same time interval. It may be warranted to investigate WMH regression from anatomic perspective in future studies. Also, because the number of participants with WMH regression is limited in our study, therefore, it might be underpowered to capture the difference between those who had WMH regression versus not.

Major strengths of the present study include the inclusion of multiple MRI markers of SVD with 4 repeated MRI scans over 14 years, a large cohort of participants with a single-center design. Furthermore, all neuroimaging data were analyzed by raters blinded to clinical information and were assessed reliably and sensitively. For instance, the use of difference images to identify incident lacunes from co-registered scans offers clear advantages over side-by-side inspection of nonregistered scans. Finally, our study has high external validity for SVD patients from general neurology clinics because, at baseline, we included all consecutive patients referred to our out-patient department with cerebral small vessel disease on neuroimaging and accompanying acute or subacute clinical symptoms.

Some limitations should be considered. First, one major limitation is the scanner change and differences in the acquisition protocol during the follow-ups, which are known to induce measurement variability.^46^ However, these differences become almost inevitable with improvements in hardware and sequence design, especially for very long follow-up studies. Second, nonparticipants were older at baseline and had a higher WMH volume compared with participants. Because our results showed that (older) patients with higher WMH burden also show more WMH progression over time, the lost-to-follow-up of these patients could have underestimated the true WMH fluctuation. Third, the number of participants with incident lacunes is limited in our study, therefore, it might be underpowered to examine the relation between vascular risk factors and incident lacunes. In addition, there is an increasing awareness for a role of genetic variants that may relate to hypertension or interaction with hypertension resulting in vulnerability to SVD.^47^ Future investigations on the genetic role in SVD progression will increase our understanding of determinants of SVD temporal dynamics. Finally, in our study, treatments for vascular risk factors and its changes over time during the follow-ups could not have been accounted for because this would result in >20 subgroups, leading to very few participants in each group. Future follow-up studies with the same scanner, MRI protocol, and more detailed, quantitive assessment of vascular risk factors, its treatment, and changes over time would be needed to validate our main findings.

In conclusion, our prospective study with serial MRI scans and quantitative assessments in a large number of elderly patients with sporadic SVD over 14 years, provides insight into the temporal dynamics of WMH and lacunes. Baseline vascular risk factors and SVD markers contributed to the long-term progression of SVD independently and synergistically. These observations may contribute to identifying those who are at risk of fast progression.

## Article Information

### Sources of Funding

This work was supported by China Scholarship Council (201706100189 to Dr Cai), the Dutch Heart Foundation (grant 2016 T044 to Dr Tuladhar), and the Netherlands CardioVascular Research Initiative: the Dutch Heart Foundation (CVON 2018-28 and 2012-06 Heart Brain Connection to Dr Tuladhar), VIDI innovational grant from The Netherlands Organization for Health Research and Development (ZonMw grant 016.126.351 to Dr de Leeuw).

### Disclosures

Dr Duering reports compensation from Sanofi for other services; compensation from F. Hoffmann-La Roche for consultant services; and compensation from Hovid Berhad for end point review committee services. The other authors report no conflicts.

### Supplemental Material

Supplemental Methods

STROBE checklist

Tables S1–S5

Figures S1–S4

## Supplementary Material



## References

[1] Wardlaw JM Smith EE Biessels GJ Cordonnier C Fazekas F Frayne R Lindley RI O’Brien JT Barkhof F Benavente OR; STandards for ReportIng Vascular changes on nEuroimaging (STRIVE v1). Neuroimaging standards for research into small vessel disease and its contribution to ageing and neurodegeneration. Lancet Neurol. 2013;12:822–838. doi: 10.1016/S1474-4422(13)70124-8 23867200 10.1016/S1474-4422(13)70124-8 PMC3714437

[2] Pantoni L. Cerebral small vessel disease: from pathogenesis and clinical characteristics to therapeutic challenges. Lancet Neurol. 2010;9:689–701. doi: 10.1016/S1474-4422(10)70104-6 20610345 10.1016/S1474-4422(10)70104-6

[3] Wardlaw JM Smith C Dichgans M. Mechanisms of sporadic cerebral small vessel disease: insights from neuroimaging. Lancet Neurol. 2013;12:483–497. doi: 10.1016/S1474-4422(13)70060-7 23602162 10.1016/S1474-4422(13)70060-7 PMC3836247

[4] Schmidt R Berghold A Jokinen H Gouw AA van der Flier WM Barkhof F Scheltens P Petrovic K Madureira S Verdelho A; LADIS Study Group. White matter lesion progression in LADIS: frequency, clinical effects, and sample size calculations. Stroke. 2012;43:2643–2647. doi: 10.1161/STROKEAHA.112.662593 22879094 10.1161/STROKEAHA.112.662593

[5] Silbert LC Nelson C Howieson DB Moore MM Kaye JA. Impact of white matter hyperintensity volume progression on rate of cognitive and motor decline. Neurology. 2008;71:108–113. doi: 10.1212/01.wnl.0000316799.86917.37 18606964 10.1212/01.wnl.0000316799.86917.37 PMC2676966

[6] Callisaya ML Beare R Phan TG Blizzard L Thrift AG Chen J Srikanth VK. Brain structural change and gait decline: a longitudinal population-based study. J Am Geriatr Soc. 2013;61:1074–1079. doi: 10.1111/jgs.12331 23796055 10.1111/jgs.12331

[7] van der Holst HM Tuladhar AM Zerbi V van Uden IWM de Laat KF van Leijsen EMC Ghafoorian M Platel B Bergkamp MI van Norden AGW. White matter changes and gait decline in cerebral small vessel disease. Neuroimage Clin. 2018;17:731–738. doi: 10.1016/j.nicl.2017.12.007 29270357 10.1016/j.nicl.2017.12.007 PMC5730123

[8] Firbank MJ Teodorczuk A van der Flier WM Gouw AA Wallin A Erkinjuntti T Inzitari D Wahlund LO Pantoni L Poggesi A; LADIS Group. Relationship between progression of brain white matter changes and late-life depression: 3-year results from the LADIS study. Br J Psychiatry. 2012;201:40–45. doi: 10.1192/bjp.bp.111.098897 22626634 10.1192/bjp.bp.111.098897

[9] Schmidt R Seiler S Loitfelder M. Longitudinal change of small-vessel disease-related brain abnormalities. J Cereb Blood Flow Metab. 2016;36:26–39. doi: 10.1038/jcbfm.2015.72 25899293 10.1038/jcbfm.2015.72 PMC4758559

[10] van Leijsen EMC de Leeuw F-E Tuladhar AM. Disease progression and regression in sporadic small vessel disease–insights from neuroimaging. Clinical Science. 2017;131:1191–1206. 28566448 10.1042/CS20160384

[11] Xia Y Shen Y Wang Y Yang L Wang Y Li Y Liang X Zhao Q Wu J Chu S. White matter hyperintensities associated with progression of cerebral small vessel disease: a 7-year Chinese urban community study. Aging (Albany NY). 2020;12:8506–8522. doi: 10.18632/aging.103154 32388497 10.18632/aging.103154 PMC7244059

[12] van Dijk EJ Prins ND Vrooman HA Hofman A Koudstaal PJ Breteler MM. Progression of cerebral small vessel disease in relation to risk factors and cognitive consequences: Rotterdam Scan study. Stroke. 2008;39:2712–2719. doi: 10.1161/STROKEAHA.107.513176 18635849 10.1161/STROKEAHA.107.513176

[13] van Leijsen EMC van Uden IWM Ghafoorian M Bergkamp MI Lohner V Kooijmans ECM van der Holst HM Tuladhar AM Norris DG van Dijk EJ. Nonlinear temporal dynamics of cerebral small vessel disease: the RUN DMC study. Neurology. 2017;89:1569–1577. doi: 10.1212/WNL.0000000000004490 28878046 10.1212/WNL.0000000000004490 PMC5634663

[14] Wardlaw JM Valdés Hernández MC Muñoz-Maniega S. What are white matter hyperintensities made of?: relevance to vascular cognitive impairment. JAHA. 2015;4. doi: 10.1161/JAHA.114.001140 10.1161/JAHA.114.001140 PMC4599520 26104658

[15] Cho AH Kim HR Kim W Yang DW. White matter hyperintensity in ischemic stroke patients: it may regress over time. J Stroke. 2015;17:60–66. doi: 10.5853/jos.2015.17.1.60 25692108 10.5853/jos.2015.17.1.60 PMC4325632

[16] van Norden AG de Laat KF Gons RA van Uden IW van Dijk EJ van Oudheusden LJ Esselink RA Bloem BR van Engelen BG Zwarts MJ. Causes and consequences of cerebral small vessel disease. The RUN DMC study: a prospective cohort study. Study rationale and protocol. BMC Neurol. 2011;11:29. doi: 10.1186/1471-2377-11-29 21356112 10.1186/1471-2377-11-29 PMC3053228

[17] Erkinjuntti T. Subcortical vascular dementia. Cerebrovasc Dis. 2002;13 Suppl 2:58–60. doi: 10.1159/000049152 11901245 10.1159/000049152

[18] Ghafoorian M Karssemeijer N van Uden IW de Leeuw FE Heskes T Marchiori E Platel B. Automated detection of white matter hyperintensities of all sizes in cerebral small vessel disease: Automated detection of white matter hyperintensities of all sizes. Med Phys. 2016;43:6246–6258. doi: 10.1118/1.4966029 27908171 10.1118/1.4966029

[19] O’Brien KR Kober T Hagmann P Maeder P Marques J Lazeyras F Krueger G Roche A. Robust T1-weighted structural brain imaging and morphometry at 7T using MP2RAGE. PLoS One. 2014;9:e99676. doi: 10.1371/journal.pone.0099676 24932514 10.1371/journal.pone.0099676 PMC4059664

[20] Long J Shelhamer E Darrell T. Fully convolutional networks for semantic segmentation. arXiv:1411.4038 [cs]. 2015;39:640–651. doi: 10.1109/TPAMI.2016.2572683 10.1109/TPAMI.2016.2572683 27244717

[21] Charidimou A Jäger HR Werring DJ. Cerebral microbleed detection and mapping: principles, methodological aspects and rationale in vascular dementia. Exp Gerontol. 2012;47:843–852. doi: 10.1016/j.exger.2012.06.008 22750456 10.1016/j.exger.2012.06.008

[22] Fazekas F Chawluk JB Alavi A Hurtig HI Zimmerman RA. MR signal abnormalities at 1.5 T in Alzheimer’s dementia and normal aging. AJR Am J Roentgenol. 1987;149:351–356. doi: 10.2214/ajr.149.2.351 3496763 10.2214/ajr.149.2.351

[23] Amin Al Olama A Wason JMS Tuladhar AM van Leijsen EMC Koini M Hofer E Morris RG Schmidt R de Leeuw F-E Markus HS. Simple MRI score aids prediction of dementia in cerebral small vessel disease. Neurology. 2020;94:e1294–e1302. doi: 10.1212/WNL.0000000000009141 32123050 10.1212/WNL.0000000000009141 PMC7274929

[24] Staals J Makin SD Doubal FN Dennis MS Wardlaw JM. Stroke subtype, vascular risk factors, and total MRI brain small-vessel disease burden. Neurology. 2014;83:1228–1234. doi: 10.1212/WNL.0000000000000837 25165388 10.1212/WNL.0000000000000837 PMC4180484

[25] Duering M Csanadi E Gesierich B Jouvent E Hervé D Seiler S Belaroussi B Ropele S Schmidt R Chabriat H Dichgans M. Incident lacunes preferentially localize to the edge of white matter hyperintensities: insights into the pathophysiology of cerebral small vessel disease. Brain. 2013;136(pt 9):2717–2726. doi: 10.1093/brain/awt184 23864274 10.1093/brain/awt184

[26] Ter Telgte A Wiegertjes K Gesierich B Marques JP Huebner M de Klerk JJ Schreuder FHBM Araque Caballero MA Kuijf HJ Norris DG. Contribution of acute infarcts to cerebral small vessel disease progression. Ann Neurol. 2019;86:582–592. doi: 10.1002/ana.25556 31340067 10.1002/ana.25556 PMC6771732

[27] Putaala J Haapaniemi E Kaste M Tatlisumak T. How does number of risk factors affect prognosis in young patients with ischemic stroke? Stroke. 2012;43:356–361. doi: 10.1161/STROKEAHA.111.635276 22052508 10.1161/STROKEAHA.111.635276

[28] Ware JE Jr Sherbourne CD. The MOS 36-item short-form health survey (SF-36). I. Conceptual framework and item selection. Med Care. 1992;30:473–483. 1593914

[29] Yirga AA Melesse SF Mwambi HG Ayele DG. Negative binomial mixed models for analyzing longitudinal CD4 count data. Sci Rep. 2020;10:16742. doi: 10.1038/s41598-020-73883-7 33028929 10.1038/s41598-020-73883-7 PMC7541535

[30] Schmidt R Enzinger C Ropele S Schmidt H Fazekas F; Austrian Stroke Prevention Study. Progression of cerebral white matter lesions: 6-year results of the Austrian Stroke Prevention study. Lancet. 2003;361:2046–2048. doi: 10.1016/s0140-6736(03)13616-1 12814718 10.1016/s0140-6736(03)13616-1

[31] Sachdev P Wen W Chen X Brodaty H. Progression of white matter hyperintensities in elderly individuals over 3 years. Neurology. 2007;68:214–222. doi: 10.1212/01.wnl.0000251302.55202.73 17224576 10.1212/01.wnl.0000251302.55202.73

[32] Gouw AA van der Flier WM Pantoni L Inzitari D Erkinjuntti T Wahlund LO Waldemar G Schmidt R Fazekas F Scheltens P Barkhof F; LADIS Study Group. On the etiology of incident brain lacunes: longitudinal observations from the LADIS study. Stroke. 2008;39:3083–3085. doi: 10.1161/STROKEAHA.108.521807 18703801 10.1161/STROKEAHA.108.521807

[33] Maniega SM Valdés Hernández MC Clayden JD Royle NA Murray C Morris Z Aribisala BS Gow AJ Starr JM Bastin ME. White matter hyperintensities and normal-appearing white matter integrity in the aging brain. Neurobiol Aging. 2015;36:909–918. doi: 10.1016/j.neurobiolaging.2014.07.048 25457555 10.1016/j.neurobiolaging.2014.07.048 PMC4321830

[34] Gons RA de Laat KF van Norden AG van Oudheusden LJ van Uden IW Norris DG Zwiers MP de Leeuw FE. Hypertension and cerebral diffusion tensor imaging in small vessel disease. Stroke. 2010;41:2801–2806. doi: 10.1161/STROKEAHA.110.597237 21030696 10.1161/STROKEAHA.110.597237

[35] Gons RA van Norden AG de Laat KF van Oudheusden LJ van Uden IW Zwiers MP Norris DG de Leeuw FE. Cigarette smoking is associated with reduced microstructural integrity of cerebral white matter. Brain. 2011;134(pt 7):2116–2124. doi: 10.1093/brain/awr145 21705426 10.1093/brain/awr145

[36] Verstynen TD Weinstein A Erickson KI Sheu LK Marsland AL Gianaros PJ. Competing physiological pathways link individual differences in weight and abdominal adiposity to white matter microstructure. Neuroimage. 2013;79:129–137. doi: 10.1016/j.neuroimage.2013.04.075 23639257 10.1016/j.neuroimage.2013.04.075 PMC3752776

[37] Kullmann S Schweizer F Veit R Fritsche A Preissl H. Compromised white matter integrity in obesity. Obes Rev. 2015;16:273–281. doi: 10.1111/obr.12248 25676886 10.1111/obr.12248

[38] Alfaro FJ Gavrieli A Saade-Lemus P Lioutas VA Upadhyay J Novak V. White matter microstructure and cognitive decline in metabolic syndrome: a review of diffusion tensor imaging. Metabolism. 2018;78:52–68. doi: 10.1016/j.metabol.2017.08.009 28920863 10.1016/j.metabol.2017.08.009 PMC5732847

[39] Zhuo Y Fang F Lu L Li T Lian J Xiong Y Kong D Li K. White matter impairment in type 2 diabetes mellitus with and without microvascular disease. Neuroimage Clin. 2019;24:101945. doi: 10.1016/j.nicl.2019.101945 31374399 10.1016/j.nicl.2019.101945 PMC6676007

[40] van Leijsen EMC Bergkamp MI van Uden IWM Ghafoorian M van der Holst HM Norris DG Platel B Tuladhar AM de Leeuw FE. Progression of white matter hyperintensities preceded by heterogeneous decline of microstructural integrity. Stroke. 2018;49:1386–1393. doi: 10.1161/STROKEAHA.118.020980 29724890 10.1161/STROKEAHA.118.020980

[41] Ling Y Chabriat H. Incident cerebral lacunes: a review. J Cereb Blood Flow Metab. 2020;40:909–921. doi: 10.1177/0271678X20908361 32126874 10.1177/0271678X20908361 PMC7181083

[42] Nasrallah IM Pajewski NM Auchus AP Chelune G Cheung AK Cleveland ML Coker LH Crowe MG Cushman WC; The SPRINT MIND Investigators for the SPRINT Research Group. Association of intensive vs standard blood pressure control with cerebral white matter lesions. JAMA. 2019;322:524. doi: 10.1001/jama.2019.10551 31408137 10.1001/jama.2019.10551 PMC6692679

[43] Maillard P Fletcher E Lockhart SN Roach AE Reed B Mungas D DeCarli C Carmichael OT. White matter hyperintensities and their penumbra lie along a continuum of injury in the aging brain. Stroke. 2014;45:1721–1726. doi: 10.1161/STROKEAHA.113.004084 24781079 10.1161/STROKEAHA.113.004084 PMC4102626

[44] Ramirez J McNeely AA Berezuk C Gao F Black SE. Dynamic progression of white matter hyperintensities in Alzheimer’s disease and normal aging: results from the Sunnybrook Dementia study. Front Aging Neurosci. 2016;8:62. doi: 10.3389/fnagi.2016.00062 27047377 10.3389/fnagi.2016.00062 PMC4805606

[45] Wardlaw JM Sandercock PA Dennis MS Starr J. Is breakdown of the blood-brain barrier responsible for lacunar stroke, leukoaraiosis, and dementia? Stroke. 2003;34:806–812. doi: 10.1161/01.STR.0000058480.77236.B3 10.1161/01.STR.0000058480.77236.B3 12624314

[46] Bordin V Bertani I Mattioli I Sundaresan V McCarthy P Suri S Zsoldos E Filippini N Mahmood A Melazzini L. Integrating large-scale neuroimaging research datasets: harmonisation of white matter hyperintensity measurements across Whitehall and UK Biobank datasets. Neuroimage. 2021;237:118189. doi: 10.1016/j.neuroimage.2021.118189 34022383 10.1016/j.neuroimage.2021.118189 PMC8285593

[47] Sargurupremraj M Suzuki H Jian X Sarnowski C Evans TE Bis JC Eiriksdottir G Sakaue S; International Network against Thrombosis (INVENT) Consortium, International Headache Genomics Consortium (IHGC). Cerebral small vessel disease genomics and its implications across the lifespan. Nat Commun. 2020;11:6285. 33293549 10.1038/s41467-020-19111-2 PMC7722866

