# Supplementary material for: Determinants and Temporal Dynamics of Cerebral Small Vessel Disease: 14-Year Follow-Up
Source: Stroke. 2022 May 4;53(9):2789–98. doi: 10.1161/STROKEAHA.121.038099 (PMC9389939; doi:10.1161/STROKEAHA.121.038099)
Supplement: Supplementary file 1 [file str-53-2789-s001.pdf]

Supplementary Materials

Supplemental Methods

Details of brain volumetry

Since we have changed FLAIR acquisition protocol during the follow-up. To minimize the effects of changes in FLAIR acquisition sequence parameters, we resampled follow-up FLAIR images to align to baseline FLAIR images using FMRIB's Linear Image Registration Tool (FLIRT), part of FSL. Thereby, all FLAIR scans have the same slice thickness and voxel size. Of note, we have used different segmentation algorithms to segment WMH between wave 1, 2, 3 and wave 4. Therefore, we evaluated the comparability of the two segmentation algorithms by using intraclass coefficient (ICC), which is a measure of how similar measurements are. Specifically, we randomly selected ~10% (n=75) of all FLAIR scans from wave 1, 2, 3, and used the 3D U-net deep learning algorithm from wave 4 to segment WMH. The derived WMH volumes were highly comparable between these two different algorithms (ICC=0.98)

Grey matter (GM), white matter (WM) and CSF probability maps were produced using SPM12 (http://www.fil.ion.ucl.ac.uk/spm/) tissue segmentation algorithm from T1 images across four waves. Additionally, we used WMH masks to correct the segmented images, since several brain regions with WMH were initially misclassified. GM, WM and CSF volumes were computed by summing all voxels belonging to that tissue class multiplied by voxel volume in ml. ICV was the sum of GM, WM and CSF volumes.

Table S1. MRI acquisition parameters.

Sequence	Feature	2006	2011	2015	2020
	Scanner	Magnetom Sonata 1.5 Tesla	Magnetom Avanto 1.5 Tesla	Magnetom Avanto 1.5 Tesla	Magnetom Prisma 3 Tesla
	Coil Channels	8 (head)	8 (head)	8 (head)	32 (head)
3D-T1	Type	MPRAGE	MPRAGE	MPRAGE	MP2RAGE
	TR (ms)	2250	2250	2250	5500
	TE (ms)	3.68	2.95	2.95	3.84
	TI (ms)	850	850	850	700/2500
	Voxel size [mm]	1.0 isotropic	1.0 isotropic	1.0 isotropic	0.85 isotropic
3D-FLAIR	TR (ms)	9000	14240	14240	5000
	TE (ms)	84	89	89	394
	TI (ms)	2200	2200	2200	180
	Voxel size [mm]	1.2×1.0×5.0	1.2×1.0×2.5	1.2×1.0×2.5	0.85 isotropic
GRE	TR (ms)	800	800	800	35
	TE (ms)	26	26	26	29.5
	Voxel size [mm]	1.3×1.0×5.0	1.3×1.0×5.0	1.3×1.0×5.0	0.9×0.9×2.0

Abbreviations: FLAIR = fluid-attenuated inversion recovery; GRE = gradient echo; MP2RAGE = magnetization prepared (2) rapid acquisition gradient echo(es); TE = echo time; TI = inversion time; TR = repetition time

Table S2: Fixed effects results for WMH progression over time as to each vascular risk factor

	Model 1		Model 2		Model 3		Model 4	
Predictors	Estimate (95% CI)	P Value	Estimate (95% CI)	P Value	Estimate (95% CI)	P Value	Estimate (95% CI)	P Value
(Intercept)	-10.15 (-16.8 – -3.53)	**0.003**	-8.90 (-15.3 – -2.51)	**0.006**	-9.09 (-15.5 – -2.68)	**0.005**	-9.41 (-15.8 – -3.00)	**0.004**
Age_baseline	0.25 (0.15 – 0.35)	**<0.001**	0.25 (0.15 – 0.34)	**<0.001**	0.25 (0.15 – 0.34)	**<0.001**	0.21 (0.11 – 0.32)	**<0.001**
Time	0.56 (0.40 – 0.71)	**<0.001**	0.58 (0.49 – 0.67)	**<0.001**	0.56 (0.45 – 0.68)	**<0.001**	0.41 (0.26 – 0.57)	**<0.001**
smoking	1.49 (-0.64 – 3.61)	0.171						
Time * smoking	0.06 (-0.13 – 0.24)	0.537						
diabetes06			1.42 (-1.62 – 4.46)	0.359				
Time * diabetes			0.12 (-0.15 – 0.39)	0.377				
Hypercholesterolemia					0.76 (-1.21 – 2.73)	0.449		
Time * hypercholesterolemia					0.08 (-0.10 – 0.25)	0.392		
hypertension							3.84 (1.68 – 6.01)	**0.001**
Time * hypertension							0.27 (0.08 – 0.45)	**0.005**

Table S3: Fixed effects results for WMH progression over time as to Fazekas groups

	**WMH**	
Predictors	**Estimate (95% CI)**	**P Value**
(Intercept)	-5.86 (-9.81 – -1.90)	**0.004**
Age	0.13 (0.07 – 0.19)	**<0.001**
Time	0.27 (0.20 – 0.35)	**<0.001**
Fazekas [moderate]	9.18 (7.85 – 10.52)	**<0.001**
Fazekas [severe]	29.21 (27.44 – 30.99)	**<0.001**
Time * Fazekas [moderate]	0.99 (0.82 – 1.16)	**<0.001**
Time * Fazekas [severe]	1.59 (1.35 – 1.83)	**<0.001**

Table S4: Fixed effects results for lacune count over time as to age and sex

	**Model 1**		**Model 2**	
Predictors	Estimate (95% CI)	*P value*	Estimate (95% CI)	*P Value*
(Intercept)	-9.13 (-10.81 – -7.45)	**<0.001**	-9.03 (-11.10 – -6.96)	**<0.001**
Age	0.08 (0.06 – 0.11)	**<0.001**	0.08 (0.05 – 0.11)	**<0.001**
Time	0.08 (0.03 – 0.14)	**0.002**	0.08 (0.03 – 0.14)	**0.003**
Sex [Female]			-0.18 (-0.95 – 0.60)	0.652
Time * Sex [Female]			0.00 (-0.03 – 0.04)	0.899

Male as the reference group.

Table S5: The effect of baseline vascular risk factors and SVD burden over time on incident lacune

	**Model 1**		**Model 2**			**Model 3**	
Predictors	Estimate (95% CI)	*P value*	Estimate (95% CI)		*P value*	Estimate (95% CI)	*P value*
(Intercept)	0.29 (0.03 – 3.30)	0.295	0.21	(0.01 – 3.57)	0.248	0.38 (0.01 – 12.08)	0.545
Age	0.98 (0.94 – 1.01)	0.191	0.99	(0.95 – 1.03)	0.432	0.97 (0.94 – 1.01)	0.168
SVD_score	1.52 (1.27 – 1.85)	**<0.001**				1.09 (0.11 – 6.17)	0.909
RF_score [1]			1.81	(0.38 – 10.00)	0.460	0.78 (0.03 – 18.17)	0.858
RF_score [>=2]			2.24	(0.53 – 11.40)	0.286	0.79 (0.04 – 15.16)	0.858
SVD_score * RF_score [1]						1.65 (0.26 – 18.11)	0.532
SVD_score * RF_score [>=2]						1.40 (0.25 – 14.18)	0.662

RF score [0] as the reference group.

RF: risk factors; CI: confidence interval

Figure S1. Identification of incident lacunes

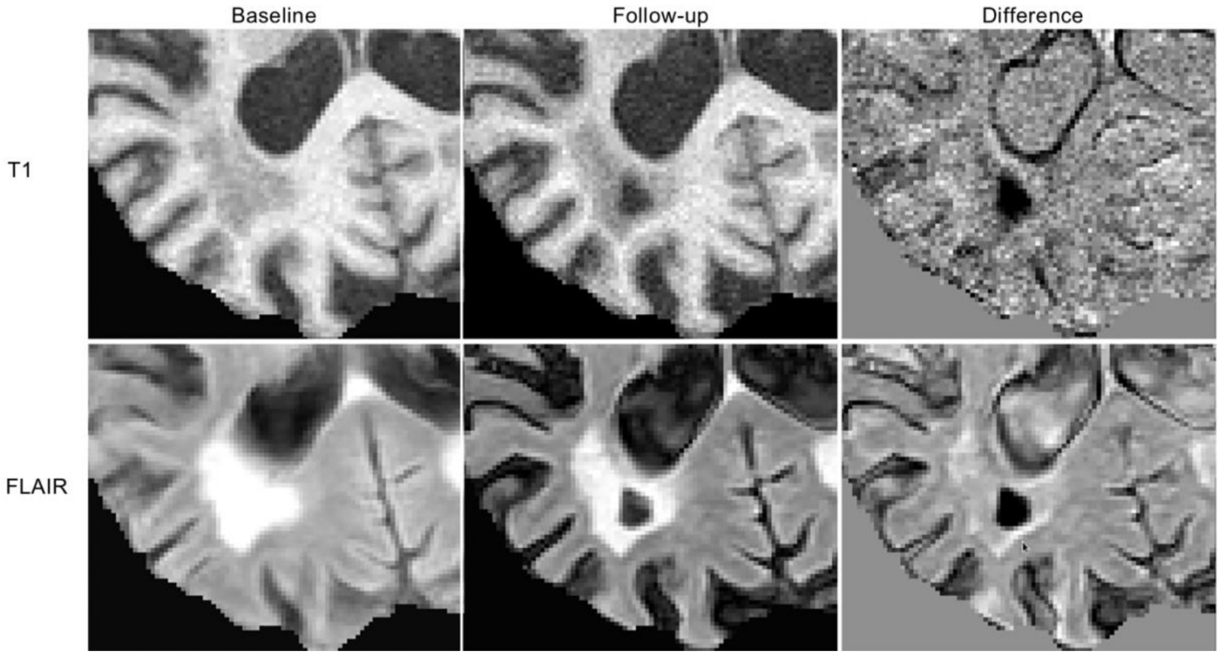

Incident lacunes were identified on T1 and FLAIR difference images created by subtracting baseline T1 and FLAIR scans from the follow-up scans.

Figure S2. WMH regression over time (n=15)

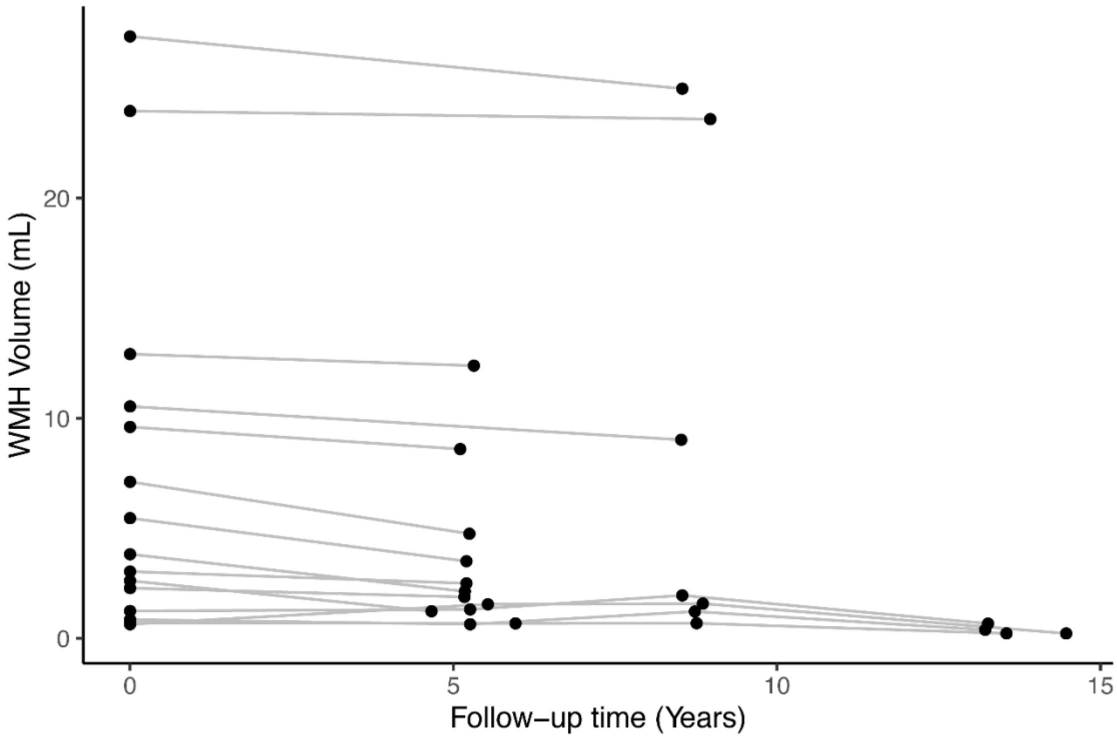

SVD participants with WMH regression for 14-years. WMH volumes for each patient with regression were plotted over time.

Figure S3. Representative FLAIR scans of WMH regression over time

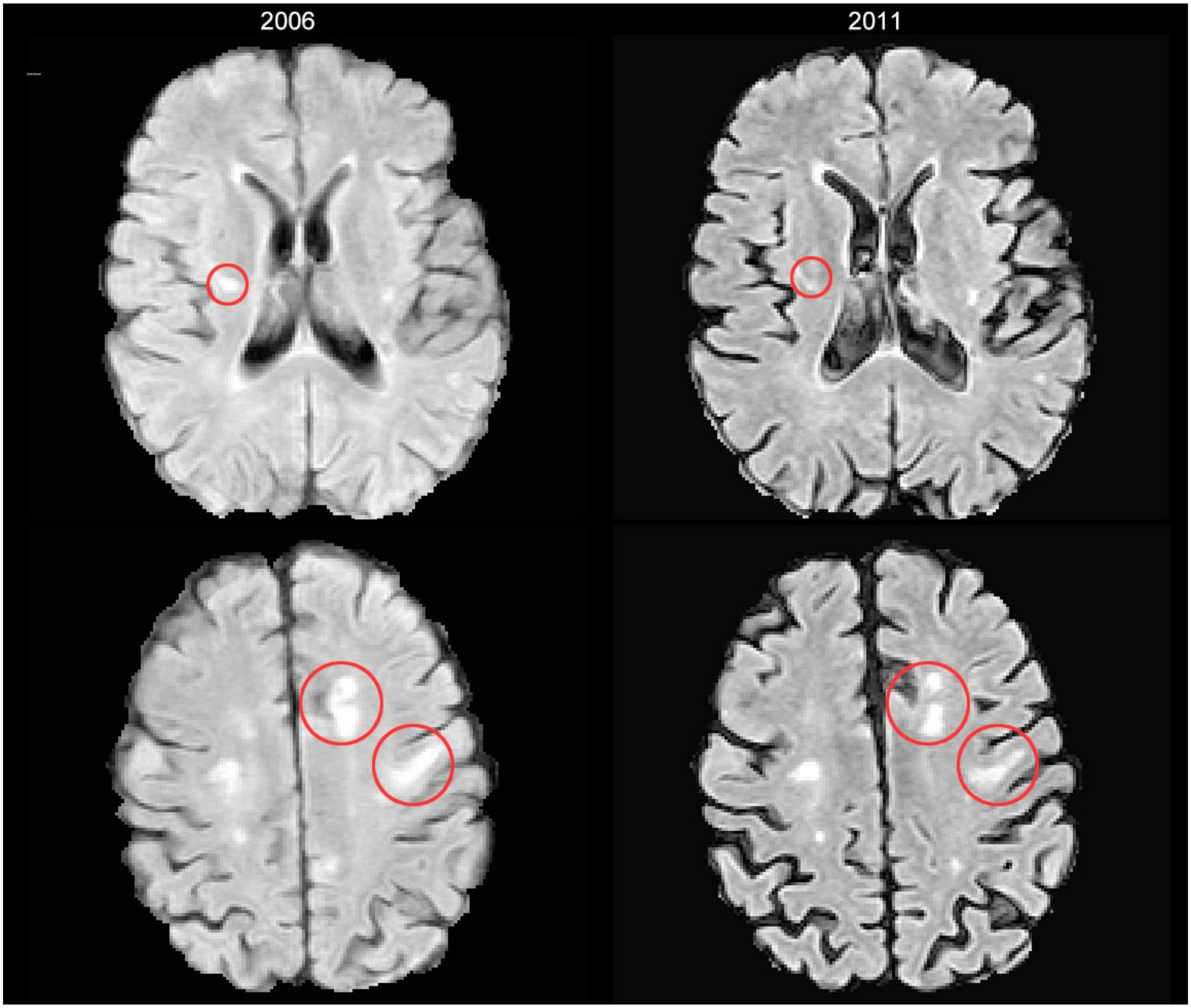

One representative participant at baseline showed WMH regression at the follow-up in 2011. The representative regions with WMH regression were enclosed by the red circle. Of note, the FLAIR scan in 2011 was registered into the native FLAIR space in 2006 to ensure that the scans from two time points were comparable anatomically with side-by-side inspection.

Figure S4. Patients with WMH regression stratified by for each time interval.

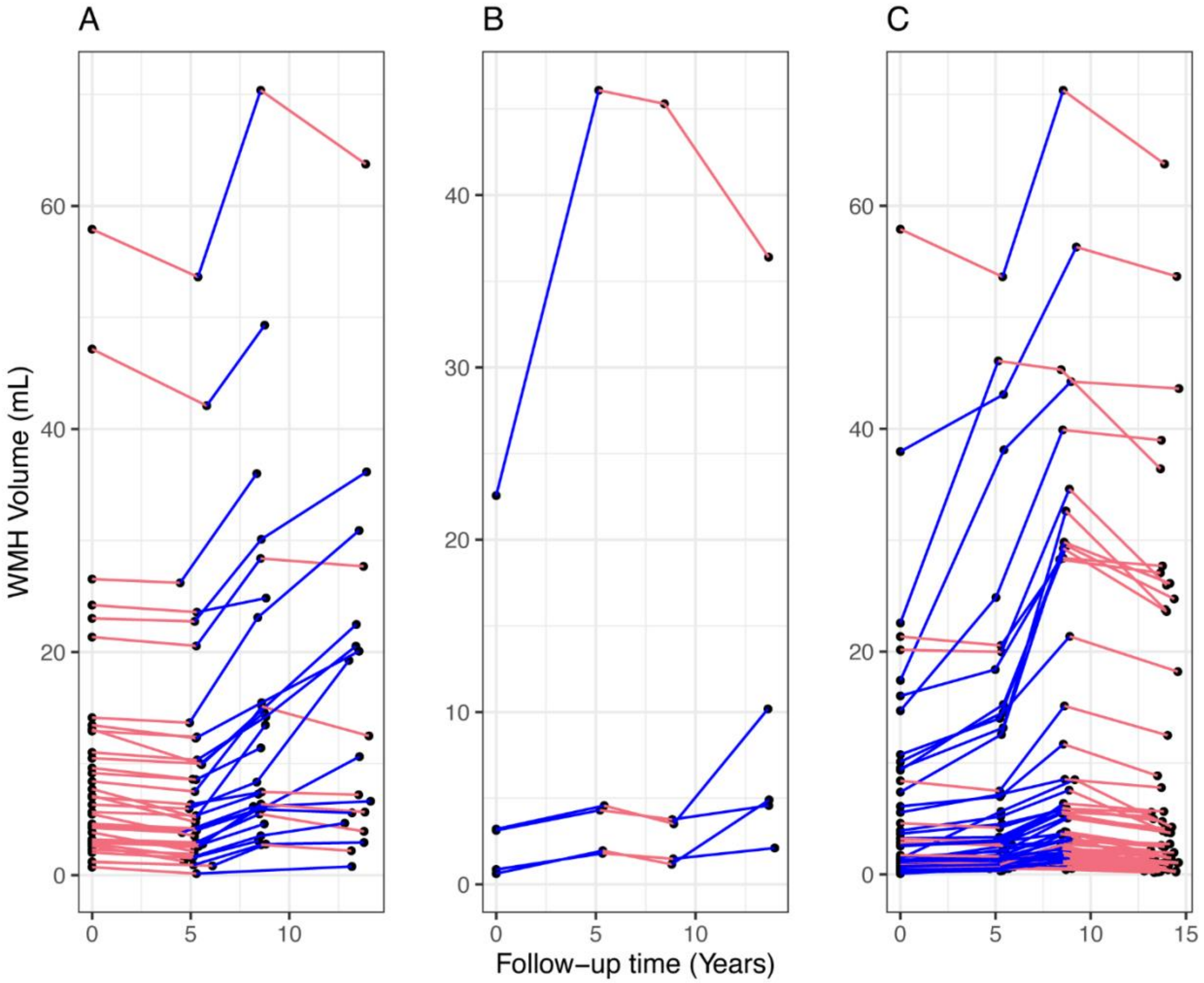

WMH regressors and progressors were labeled by red and blue for each interval, respectively. (A) 38 WMH regressors for between year 0 and 5 (2006-2011). Among these 38 participants, 9 had 2 MRI scans only and showed WMH regression, while all the remaining 29 (76%) (with >2 scans) showed overall progression in 14-year follow-up. (B) 5 WMH regressors between year 5 and 10 (2011-2015) and all had 4 MRI scans, while they all showed an overall WMH progression. (C) 61 WMH regressors between year 10 and 15 (2015-2020) and 1/60 had 3/4 MRI scans, respectively. Of note, during the 14-year follow-up, 4 showed overall WMH regression, while all the others 57 (93%) showed an overall WMH progression.

STROBE Statement—Checklist of items that should be included in reports of **cohort studies**

	Item No	Recommendation	Page No.
**Title and abstract**	1	(a) Indicate the study's design with a commonly used term in the title or the abstract	P1, line4-6
		(b) Provide in the abstract an informative and balanced summary of what was done and what was found	P1, line8-19
**Introduction**			
Background/rationale	2	Explain the scientific background and rationale for the investigation being reported	P2, line 30-42
Objectives	3	State specific objectives, including any prespecified hypotheses	P2, line 44-48
**Methods**			
Study design	4	Present key elements of study design early in the paper	P3, line53-70
Setting	5	Describe the setting, locations, and relevant dates, including periods of recruitment, exposure, follow-up, and data collection	P3, line53-70
Participants	6	(a) Give the eligibility criteria, and the sources and methods of selection of participants. Describe methods of follow-up	P3, line53-70
		(b) For matched studies, give matching criteria and number of exposed and unexposed	NA
Variables	7	Clearly define all outcomes, exposures, predictors, potential confounders, and effect modifiers. Give diagnostic criteria, if applicable	P6-7, line 127-170
Data sources/ measurement	8*	For each variable of interest, give sources of data and details of methods of assessment (measurement). Describe comparability of assessment methods if there is more than one group	P4-5, line 77-121
Bias	9	Describe any efforts to address potential sources of bias	P4, line 85-86;<br>P7, 167-170; supplemental methods,
Study size	10	Explain how the study size was arrived at	P3, line 65-70
Quantitative variables	11	Explain how quantitative variables were handled in the analyses. If applicable, describe which groupings were chosen and why	P6, line 127-142
Statistical methods	12	(a) Describe all statistical methods, including those used to control for confounding	P6-7
		(b) Describe any methods used to examine subgroups and interactions	P6, line148-151
		(c) Explain how missing data were addressed	P7, line 167-170
		(d) If applicable, explain how loss to follow-up was addressed	P6, line 134-136
		(e) Describe any sensitivity analyses	P7, 153-155
**Results**			
Participants	13*	(a) Report numbers of individuals at each stage of study—eg numbers potentially eligible, examined for eligibility, confirmed eligible, included in the study, completing follow-up, and analysed	P7, line 172-176
		(b) Give reasons for non-participation at each stage	P3, line 65-68
		(c) Consider use of a flow diagram	Figure 1.

Descriptive data	14*	(a) Give characteristics of study participants (eg demographic, clinical, social) and information on exposures and potential confounders	Table 1
		(b) Indicate number of participants with missing data for each variable of interest	P7, line 167-170
		(c) Summarise follow-up time (eg, average and total amount)	P8, 172-176
Outcome data	15*	Report numbers of outcome events or summary measures over time	P8, 172-176
Main results	16	(a) Give unadjusted estimates and, if applicable, confounder-adjusted estimates and their precision (eg, 95% confidence interval). Make clear which confounders were adjusted for and why they were included	P8-9
		(b) Report category boundaries when continuous variables were categorized	P8, 194-197
		(c) If relevant, consider translating estimates of relative risk into absolute risk for a meaningful time period	NA.
Other analyses	17	Report other analyses done—eg analyses of subgroups and interactions, and sensitivity analyses	P8; line187-188; line195-196
**Discussion**			
Key results	18	Summarise key results with reference to study objectives	
Limitations	19	Discuss limitations of the study, taking into account sources of potential bias or imprecision. Discuss both direction and magnitude of any potential bias	P10, 283-297
Interpretation	20	Give a cautious overall interpretation of results considering objectives, limitations, multiplicity of analyses, results from similar studies, and other relevant evidence	P10-11
Generalisability	21	Discuss the generalisability (external validity) of the study results	P12, line 280-282
**Other information**			
Funding	22	Give the source of funding and the role of the funders for the present study and, if applicable, for the original study on which the present article is based	See title page.

*Give information separately for exposed and unexposed groups.

Note: An Explanation and Elaboration article discusses each checklist item and gives methodological background and published examples of transparent reporting. The STROBE checklist is best used in conjunction with this article (freely available on the Web sites of PLoS Medicine at http://www.plosmedicine.org/, Annals of Internal Medicine at http://www.annals.org/, and Epidemiology at http://www.epidem.com/). Information on the STROBE Initiative is available at http://www.strobe-statement.org.
